# Implementation of the Expert Nursing Standard: Caregivers’ Oral Health Knowledge

**DOI:** 10.3390/geriatrics9050112

**Published:** 2024-09-03

**Authors:** Ina Nitschke, Felix Schulz, Elmar Ludwig, Julia Jockusch

**Affiliations:** 1 Gerodontology Section, Department of Prosthetic Dentistry and Materials Science, Leipzig University, Liebigstraße 12, 04103 Leipzig, Germany, f.schulz@medizin.uni-leipzig.de (F.S.); 2 Dental Office, Neue Straße 115, 89073 Ulm, Germany; elmar_ludwig@t-online.de; 3 University Research Priority Program “Dynamics of Healthy Aging”, University of Zurich, CH-8050 Zurich, Switzerland

**Keywords:** expert nursing standard, caregiver, oral health knowledge, oral health education, long-term care, nursing

## Abstract

The promotion of oral health in nursing care is essential for preventing oral diseases and maintaining health in elderly vulnerable populations. There is a need for standardized guidelines and education. The aim of this study was to collect data on the attitudes and hopes of caregivers regarding the implementation of the German Expert Nursing Standard “Promotion of Oral Health in Nursing” (GENS-POHN) and to evaluate their oral healthcare knowledge before implementation. A cross-sectional study was conducted in five different care settings in Germany. A self-administered questionnaire was used to collect data on the attitudes and hopes of nursing assistants regarding the GENS-POHN. Oral healthcare knowledge was evaluated before implementation. Most participants had a positive attitude towards the GENS-POHN and hoped that its implementation would lead to greater safety and competence in daily oral healthcare tasks. Few participants currently use screening or assessment instruments for oral care. There is a need for further education and training, as well as the development and implementation of standardized guidelines and tools for screening and assessment, in oral care. The GENS-POHN as an expert standard could be made accessible to an international audience by translating it into other respective national languages, thereby enhancing its usability for a wider range of users.

## 1. Introduction

With age, the prevalence of physical or mental limitations increases. These often lead to frail individuals relying on assistance for activities of daily living, as well as their care [1]. According to the United Nations, the global population of individuals aged 65 years or older is projected to more than double, increasing from 761 million in 2021 to 1.6 billion, by 2050. Furthermore, the number of people aged 80 years or older is expected to grow even more rapidly, tripling from 143 million in 2019 to 426 million by 2050. This dramatic rise underscores a significant worldwide increase in the number of older adults who may require long-term care [2,3]. In Germany, 4.96 million people required long-term care in December 2021, as indicated by the Long-Term Care Insurance Act (Pflegeversicherungsgesetz SGB XI) [4]. As the number of people in need of care increases, so do the demands on their supportive environment, e.g., caregivers or relatives. This highlights a growing demand for long-term care worldwide.

In response to these evolving needs, the German Nursing Quality Assurance Network (Deutsches Netzwerk für Qualitätsentwicklung in der Pflege, DNQP) aims to enhance nursing care through the development of best practices and expert nursing standards in all areas of nursing [5]. The DNQP’s objectives are the following:

1. Promote and develop best practices: Develop and implement high-quality nursing standards to improve care across various settings.
2. Standardize care: Establish inter-professional expert nursing standards that serve as benchmarks for quality development in nursing care [6].
3. Facilitate implementation: Provide guidance on adapting and applying these standards to meet the specific needs of different care facilities.

The expert nursing standard makes an essential contribution to uniform quality development. Nevertheless, there can be considerable differences among facilities, as the standards define basic principles and, above all, framework conditions. However, adaptation is the responsibility of the respective facility, which then has to manage their implementation [7].

The oral health of people in need of long-term care often deteriorates because the maintenance of sufficient oral and denture hygiene by the patients themselves or by caregivers is frequently not guaranteed. This results in primary oral consequences such as an increase in the prevalence of root caries and gingival recession [8]. The resulting loss of teeth may in turn have a strong negative impact on quality of life as well as nutrition [9]. As oral and denture hygiene declines, the risk of general disease increases; edentulous patients who wear their dentures at night have an increased risk of pneumonia [10].

Interactions between oral health and general diseases are known, e.g., between periodontitis and diabetes mellitus, rheumatoid arthritis, cardiovascular disease, Parkinson’s disease, psoriasis, respiratory infections, and Alzheimer’s disease [11], although no direct cause–effect relationship has yet been demonstrated. In addition to age-specific changes, a decrease in oral and denture hygiene ability, and interactions with systemic and oral diseases, side effects of polypharmacy, e.g., through an increase in oral dryness and thus an increased risk of caries, periodontitis, and candidiasis [12], are added as potential factors for deteriorating oral health in old age.

Awareness of dental problems in nursing has been sufficiently described [6,13,14]. The existing assessment tools that are used internationally, such as the RAI-MDS (Resident Assessment Instrument–Minimum Data Set), cannot reliably depict the status of care recipients regarding oral health [15]. Several studies have shown that a lack of time and the lack of theoretical and practical knowledge of nurses lead to insufficient implementation of oral and prosthetic care protocols [13,14]. Therefore, it is demanded that the interface between nursing and dentistry is improved and further developed in the education, training, and continuing education of nurses [16,17,18].

The literature has shown that interprofessional collaboration between dentistry and nursing improves oral health education by integrating comprehensive oral assessments into traditional examinations, which improves clinical competence, increases dental referrals, and enhances the competence of the healthcare workforce. This approach promotes better patient care and addresses important public health issues by improving access to and management of oral health [19].

Another instrument to improve the interface between nursing and dentistry can be cooperation contracts financed by statutory health insurance [20]. Thus far, these contracts are concluded only between inpatient care facilities (§71(2) SGB XI) and dentists according to the conditions of the Association of Statutory Health Insurance Dentists of the respective federal state. The cooperation contracts enable dentists to better communicate and present oral situations to caregivers. The dentists are financially remunerated through these cooperation contracts, but not the care facilities [6]. In addition to cooperative agreements, there are other coordinated forms of cooperation or cooperative agreements that provide structured dental care for the residents of institutional care facilities.

Since 2019, the German Expert Nursing Standard “Promotion of Oral Health in Nursing” (GENS-POHN) has been developed in cooperation with the German Dental Association, the German Society for Geriatric Dentistry, and the German Association of Dentistry for People with Disabilities or Special Medical Support Needs [21]. From September 2021 to June 2022, model implementation of the expert standard took place in 30 facilities for inpatient geriatric care, inpatient care for younger people, outpatient care, and hospital care to obtain findings on the practical suitability and acceptance of the GENS-POHN [5].

The GENS-POHN is a quality instrument in the German healthcare system developed by the German Network for Quality Development in Nursing (DNQP). It defines the requirements for promoting oral health in care recipients by including recommendations and measures for the prevention of oral health problems, the early detection of oral diseases, and ensuring adequate care, and serves as a guide for nursing professionals.

The GENS-POHN must be individually adapted to the requirements of each facility. As a general template, it is just as applicable to a pediatric oncology ward as it is to a long-term care facility.

### Characteristics of the Expert Standards

▪ Target audience: The standard is aimed at nursing professionals responsible for the oral health of care recipients.
▪ Holistic approach: The standard considers not only the specific oral care needs of care recipients but also integrates preventive measures to prevent oral health problems.
▪ Collaboration with other healthcare professions: It emphasizes the importance of interprofessional collaboration, especially with dentists and other relevant professionals, to ensure comprehensive oral healthcare.
▪ Evaluation criteria: The standard contains criteria by which the implementation and effectiveness of measures can be assessed.

The application of this expert standard aims to promote the oral health of care recipients, identify existing problems early, and ensure high-quality care in the field of oral health [5]. A more detailed description of the GENS-POHN can be found in Supplementary File S1.

In general, the term “standard” originates from the international discussion between the World Health Organization (WHO), International Council of Nurses (ICN), and European Nursing Quality Assurance Network (Euro QUAN). It reflects the binding and far-reaching mandate associated with an instrument. Nursing standards specify the objectives and quality level of complex nursing tasks, as well as the scope for action and alternatives, and are suitable for nursing activities with a high level of interaction. Therefore, standards should not be mistaken for procedures, which are based on precise descriptions of procedures, technical instructions, or instructions on hygiene. In conjunction with medical guidelines and the quality instruments of other professional groups, the expert standard forms the basis for the sustainable implementation of nursing tasks and the basis for interprofessional cooperation in nursing [22].

This study aims to present the situation in different care settings before the implementation of the GENS-POHN. For this purpose, caregivers were interviewed on the following aspects: (a) Knowledge of the facility’s processes, procedures, and cooperation; (b) attitudes and hopes of nurses regarding the implementation of the GENS-POHN and their assessment of the structural criteria; (c) oral health and oral/denture care; and (d) knowledge of oral health and oral/denture healthcare.

## 2. Materials and Methods

### 2.1. Design

The methodological procedure for the development of expert standards in nursing comprises the four stages of development, consensus, implementation, and updating, each with its own procedural steps. Development and implementation are coordinated by the German Network for Quality Development in Nursing (DNQP). The description of the methodological approach is only available in German [22].

The GENS-POHN is currently only available in German for German-speaking countries. However, it represents a concept that, once translated into other languages, could also be used in different countries. As the extensive literature on the GENS-POHN is commercially available, the authors cannot offer a full English version of the GENS-POHN in this paper. The DNQP is affiliated with the University of Osnabrück and finances the development of new care standards from the sale of books on the already-implemented care standards. In addition to new care topics, the sale of the books also finances the adaptation of the already-implemented care standards to the new findings for care. However, it would be possible for interested parties from other countries to work together with the DNQP on adaptations to other languages.

A translation of the publicly available summary of the GENS-POHN by the group of authors based on the original document can be found in Supplementary File S1.

The data of this analysis are part of the accompanying cross-sectional dental study for the implementation of the GENS-POHN [5].

This manuscript was written following the STROBE checklist.

### 2.2. Study Setting and Sampling

#### Concept of Model Implementation and Selection of Facilities by the DNQP

Each expert standard is implemented in approximately 25 different nursing and healthcare facilities following consensus. This model implementation serves to test its acceptance and practicality. Furthermore, insights are sought into the prerequisites significant for the sustainable introduction of the expert standard in stationary nursing facilities, outpatient care services, and hospitals. A criteria-led process for selecting implementation sites, a program for professional advice and scientific support during the implementation phase, and a conceptual framework in the form of a multi-stage phase model are available for the model’s introduction of expert standards [22].

The implementation concept developed by the DNQP is in line with the key concepts of numerous international implementation models [23]. A balanced distribution is aimed for different types of facilities, a wide range of involved departments, and patient or resident groups with different nursing needs. Consideration is also given to regional distribution aspects of the facilities [22].

Calls for applications are made within the framework of the consensus conference through specialized press and the German Nursing Council. All interested facilities receive information about the selection criteria and the progress of the implementation project through this method paper and reports of completed model implementations [22].

**Figure 1:**
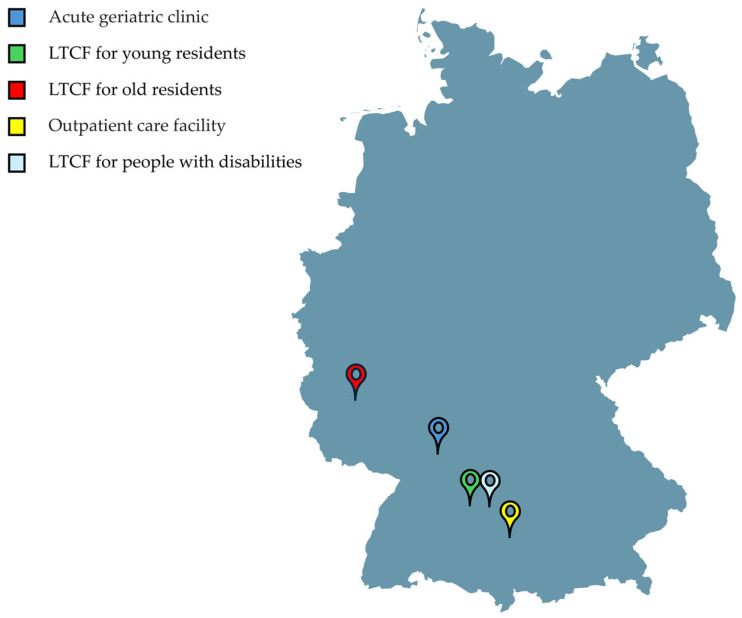
The geographical setting of the different care facility settings in Germany (LTCF—long-term care facility).

As part of the model implementation of the GENS-POHN in German facilities providing inpatient care, outpatient care, and hospital care, five facilities were monitored as part of this accompanying dental study (Figure 1). The facilities included one facility from each of the five possible settings: acute geriatric care in hospitals; outpatient care facilities; long-term care facilities (LTCFs) for younger people in need of care; LTCFs for older people in need of care; and LTCFs for people with disabilities to which the authors have had prior access due to dental cooperation with these institutions. The various institutions were interested in implementation for their internal requirements, and therefore also sought scientific collaboration with the dentist in charge of their institution and the research team.

Within the framework of the accompanying dental study, both the facility managers and the caregivers were interviewed before implementation of the GENS-POHN.

- Facility managers were interviewed by the study dentist using a standardized questionnaire.
- Caregivers were interviewed in writing using a standardized questionnaire at T0. Afterwards, they received 6 h of training in oral and denture hygiene (3 h theoretical and 3 h practical training) in an intervention by a dentist experienced in gerostomatology (not the study dentist). A second written survey of caregivers was conducted three months after implementation of the GENS-POHN (T1).

### 2.3. Inclusion Criteria

Within each of the five exemplary facilities, caregivers with different professional designations were included as participants in the study. The selection of participants was not purposive and depended on which staff members were available at the time of the initial survey and training. Participants were included based on their nursing profession as defined by the current German Classification of Occupation 2010 (revised version 2020) [24,25] (Supplementary File S2). These professional titles are assigned to the following levels of education:

▪ Level A—Head of nursing;
▪ Level B—Nursing specialists (including unit managers);
▪ Level C—Nursing assistants;
▪ Level D—Semi-skilled labor nurses;
▪ Level E—Trainees.

Not all of these nursing professions routinely carry out oral care activities, which are mostly provided by caregivers of levels C and D.

If referring to all nursing participants in general in this study, the term caregivers will be used.

All caregivers involved in the daily oral healthcare of residents were included in the study. It was up to the facilities to select the staff who met these criteria. This also applied to trainees who were involved in oral healthcare on a day-to-day basis and unit managers who fulfilled a control function and could influence the oral health of those in need of care by introducing care processes. The respondent had to be proficient in the German language so that they were able to understand and answer the questionnaire. No other exclusion criteria were defined.

### 2.4. Data Source

The measurement of caregivers’ attitudes towards the GENS-POHN in this study was realized using questionnaires. The answers to the questions were recorded using Likert scales or bi-polar rating scales. Attitudes were defined as positive or negative evaluations, attitudes, or feelings towards the introduction of the GENS-POHN in the everyday work of nursing staff. Attitude generally refers to a person’s disposition towards the surrounding reality, which shapes their behavior. Attitudes are influenced by external factors, such as sociocultural influences, and internal factors, such as personal experiences. In addition, attitudes can be influenced by information from other people, by observations, or by the mass media [26].

For this purpose, the caregivers were presented with questionnaires containing statements or questions aimed at attitudes. Then, the caregivers rated these statements on a scale (e.g., from “strongly agree” to “strongly disagree”, or from 1 to 7, with 4 being the neutral point).

Socio-demographic characteristics were queried, such as age (in years), gender (categories: male, female), period of training, and position in the facility (categories: unit manager, nursing specialist, nursing assistant, semi-skilled labor nurse, trainee, others). The period of training is defined as the entire period of education and training; therefore, it includes not only vocational training but also continuing education.

Further questionnaire sections examined (A) caregivers’ knowledge of the facility’s processes, procedures, and facility cooperation with dentists; (B) attitudes and hopes of nurses regarding implementation of the GENS-POHN and their assessment of the structural criteria; (C) oral health and oral/denture care in practice and caregivers’ awareness; and (D) theoretical knowledge of oral health and oral healthcare. For details, refer to Supplementary File S3.

### 2.5. Statistical Evaluation

The statistical analysis of all data was purely descriptive. The absolute and relative frequencies were evaluated for the qualitative parameters. Median/quartiles and range as well as mean and standard deviation were used for the quantitative parameters.

On the advice of the biometric consultant, a sample size calculation was not required from a biometric point of view; on the one hand, this is not a randomized clinical study, and on the other hand, no input data on the topic under investigation were available in the literature.

The analysis was carried out using SPSS version 27.0 [27].

### 2.6. Ethical Considerations

This study was approved by the competent ethics committee of the University of Leipzig, Germany (number: 318/21-ek). All participants or their legal representatives provided written informed consent.

## 3. Results

A total of 79 participants (mean age: 37.5 ± 13.1, median 34 (range 16–63 years) (female n = 64, 82.1%; mean age 36.6 ± 13.1 years, median age 32.5 years (range 16–62 years); male: n = 14, 17.9%; mean age 41.9 ± 12.9 years, median age 42 years (range 26–63 years); one participant without age and gender indication), eight participants without age indication) from five different settings (acute geriatric clinic, LTCF for young residents, LTCF for old residents, outpatient care facility, LTCF for people with disabilities) in Germany were included in the analysis. As the selection of participants depended on which employees were available at the time of the first survey and training, the response rate of the participants for the presented data was 100%.

Socio-demographic characteristics stratified by setting and in total are shown in Table 1. Participants predominantly included nursing assistants (n = 39, 49.4%) who had been educated for 1 or 2 years.

### 3.1. Part A—Knowledge of the Facility’s Processes, Procedures, and Cooperation

In the first part, all participants were asked about common procedures within their facility (Table 2). Not all employees knew what cooperation existed within their facility, and what procedures and admission processes were considered standard, as they were not responsible for contracts and thus did not know which cooperations had been concluded. The information provided by the facility managers was set as the gold standard. Therefore, a discrepancy between the answers of study participants and the answers of facility managers was observed (Table 2).

If the statement of the facility managers is taken as the gold standard, then, based on all questions asked (n = 7) and all answers given by the caregivers (n = 537)—excluding the answers of the caregivers of the LTCF for people with disabilities (n = 17)—to the question “Do you have one or more cooperation agreements with a dentist?” with the facility manager’s answer of “do not know”, 268 questions (49.9%) were answered correctly by the caregivers. Conversely, this means that about half of all caregivers did not know which processes were applied within their care facility (Table 2).

In the facilities themselves, there were no nursing specialists appointed as oral healthcare managers. Also, no facilities had held any further training courses on oral health in the last two years. Except for the LTCF for people with disabilities, all facilities conducted inquiries within the admission interview to determine what future medical care should be provided for the patient/resident. For dental care, this was carried out in two of the five settings. No dental examinations were arranged at the time of residents’/patients’ admission in any setting. However, except in acute geriatric care, the need for nursing support to promote oral healthcare is at least partially assessed in all areas (Table 2).

### 3.2. Part B—Attitudes and Hopes of Caregivers Regarding Implementation of the GENS-POHN and Their Assessment of the Structural Criteria

**Figure 2:**
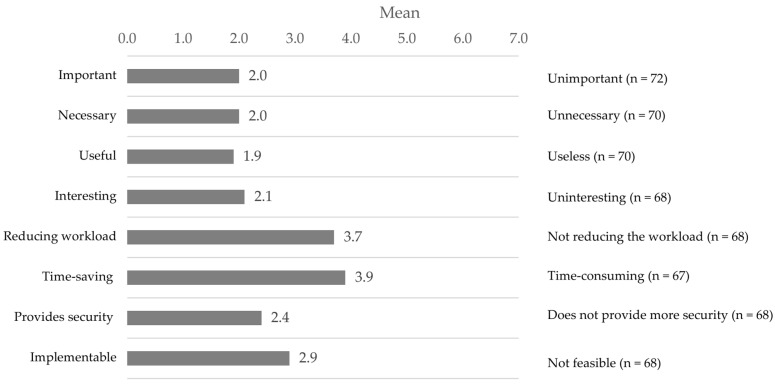
Assessment of the attitudes of the caregivers (mean scores; 4 = neutral attitude; the lower the score the more positive the attitude) towards implementation of the German Expert Nursing Standard “Promotion of Oral Health in Nursing”.

The participants predominantly had a positive attitude towards the GENS-POHN. They considered this expert nursing standard important, necessary, and useful. They predominantly assumed that it was also interesting and contributed to patient safety. A neutral attitude was taken regarding work facilitation and timesaving (Figure 2).

**Figure 3:**
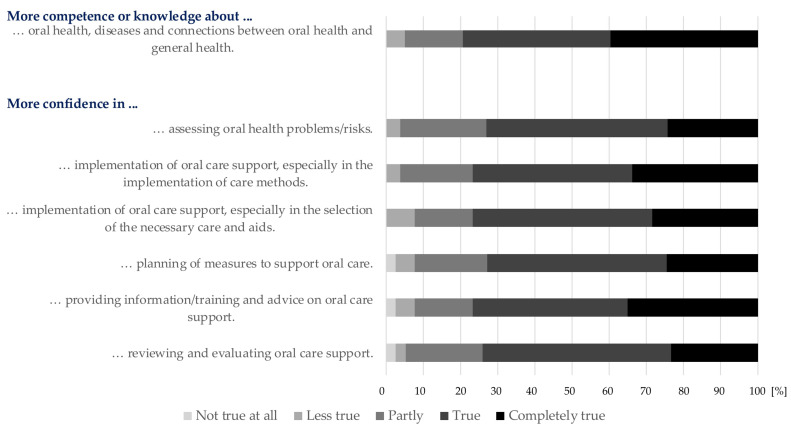
Hopes of caregivers regarding implementation of the German Expert Nursing Standard “Promotion of Oral Health in Nursing”.

Overall, most caregivers (n = 62, 78.4%, the category “completely true” or “true”) hoped that implementing the GENS-POHN would lead to greater safety and competence in their daily oral healthcare tasks, as well as to greater knowledge and certainty regarding oral health and the assessment of oral health problems and risks (Figure 3).

**Figure 4:**
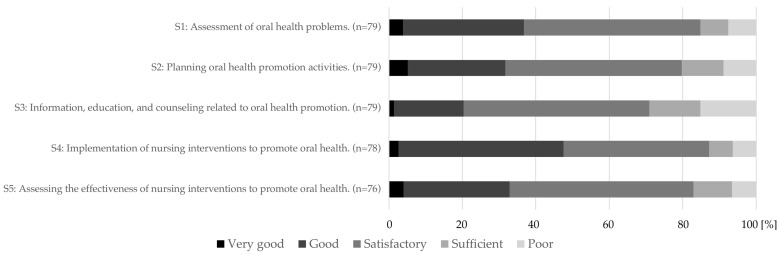
Self-assessment of caregivers on knowledge and understanding of the structural criteria (S1–S5) [22] of the new expert standard for the promotion of oral health in nursing. S1 to S5: Structural criteria of the German Expert Nursing Standard “Promotion of Oral Health in Nursing” [5].

Most participants assessed their knowledge of the structural criteria relevant to the GENS-POHN as satisfactory (standard deviation good to sufficient) (Figure 4). Consequently, there is potential for improvement through the implementation of training programs.

Most of the participants stated that they currently did not work with either a screening instrument (no screening instrument available n = 57, 73.1%) or an assessment instrument (no assessment instrument available n = 56, 72.7%) in oral care. Approximately 18.2–19.2% (n = 14 or n = 15) stated that they did not know whether such instruments were used in their institution.

### 3.3. Part C—Oral Health and Oral/Denture Care in Practice and Caregivers’ Awareness

**Figure 5:**
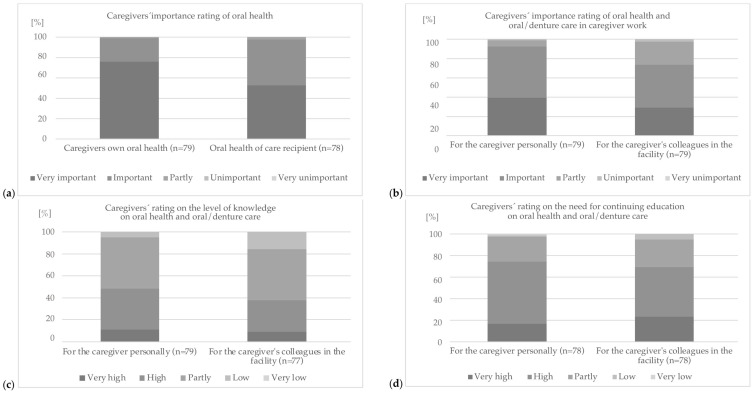
Assessment of (**a**) importance of caregivers’ own oral health and the oral health of residents/patients, (**b**) the importance of oral health and oral/denture care in caregiver work for the caregivers personally compared to what the caregivers think how important this is to their colleagues at the facility, (**c**) level of knowledge, and (**d**) need for continuing education on oral health and oral/denture care for the caregivers personally and compared to what caregivers think of their colleagues’ knowledge or need for continuing education (LTCF—long-term care facility).

Participants reported that, on average, 7.8 ± 2.4 patients/residents required assistance with oral and denture hygiene per workday at their facility (median 8, range 0–10). Almost all caregivers rated the oral health of patients/residents as important or very important (Figure 5a). However, when asked how they think their colleagues at the facility would rate the importance of oral and denture hygiene care, about 73.4% assumed that their colleagues rated this as important or very important (Figure 5b).

Overall, it was shown that the caregivers considered their own oral health as more important than that of their patients/residents (Figure 5a). Similarly, they assumed that they personally placed a higher value on oral and denture hygiene for the patient/resident than their colleagues at the facility in which they worked (Figure 5b). At the same time, they rated their own level of knowledge of oral health and oral/denture hygiene care as slightly better than the level of knowledge of their colleagues at the facility (Figure 5c). Consequently, the need for further training was also rated as very high or high by most caregivers. However, the personal need for further training was rated lower than that of the entire staff of the facility (Figure 5d).

### 3.4. Part D—Assessment of Theoretical Knowledge of Oral Health and Oral/Denture Healthcare

**Figure 6:**
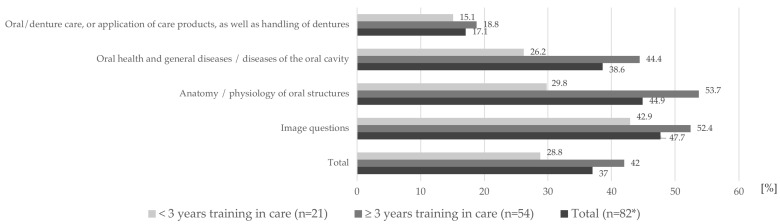
Percentages of correct answers to MC (multiple-choice; * n results from multiple answers possible) questions regarding the theoretical knowledge of caregivers on oral health and oral/denture healthcare in total and stratified in four categories (The period of training is defined as the entire period of vocational training and continuing education).

The theoretical knowledge of the caregivers in the written questionnaire as a whole and of the caregivers categorized according to the duration of their training in nursing can be rated as poor. Questions oriented to practical activities (questions on oral/denture care or application of care products, as well as handling of dentures) were only answered correctly by a very low number of respondents (correct answers: in total 17.1%; <3 years of training in care 15.1%; ≥3 years of training in care 18.8%). The best results (42.9–52.4% correct answer rate) were achieved with questions that depicted an oral situation. The difference between caregivers with three or more years of training in nursing and caregivers with less than three years of training in nursing was particularly evident in the theoretical question areas on “anatomy/physiology of oral structures” (correct answers: in total 44.9%; <3 years of training in care 29.8%; ≥3 years of training in care 53.7%) and “oral health and general diseases/diseases of the oral cavity” (correct answers: in total 38.6%; <3 years of training in care 26.2%; ≥3 years of training in care 44.4%), while the questions on the topic of complex “oral/denture care, or application of care products, as well as handling of dentures” were answered almost equally poorly (see above). Overall, a high theoretical knowledge deficit was noted in all settings and at all levels of qualification (Figure 6).

## 4. Discussion

The present study provides insights into the oral healthcare practices and attitudes of nursing staff in various settings in Germany, as well as their perceptions of the GENS-POHN [28].

### 4.1. Levels of Responsibility in Oral Healthcare

There are different levels of responsibility for daily oral health in nursing care in Germany in which personnel of different levels of education (please refer to the Section 2 for the levels of education) are involved [24,25]:

- Responsibility level I: The daily oral and denture care of the resident/patient is often performed by nursing assistants with one to two years of training and by semi-skilled labor nurses who are often only trained for a few weeks (education levels C and D).
- Responsibility level II: The nursing specialist (usually 3-year-trained nursing specialists) checks daily oral and denture care (education level B).
- Responsibility level III: The head of nursing, who, together with the management of the facility, organizes cooperation with the dentist (education level A).

Thus, there is an inverse structure in the level of responsibility compared to the level of education, i.e., the nurses with the least education and consequently the least expertise are predominantly responsible for the oral health of the residents of care facilities.

### 4.2. Findings and Concerns

The findings of this study reveal that nursing staff, predominantly nursing assistants with 1 or 2 years of education (level C), are responsible for the daily oral healthcare in most of the included facilities. None of the facilities had appointed oral healthcare managers or conducted training courses on oral health in the last two years, which is concerning since training programs for nursing staff are known to significantly improve oral healthcare practices in LTCFs [17,29,30]. Moreover, the lack of dental examinations when people are admitted to the facility and the absence of screening or assessment tools underline the urgent need to improve oral healthcare practices.

### 4.3. Training and Collaboration

The responsibility for further training must be assumed and demanded by the head of nursing (level A) and the facility’s management. However, this does not prevent nurses from seeking further knowledge on their own, such as consulting their own dentist or the facility’s dentist. Dentists are often receptive when caregivers show interest in residents’ oral care, and such support is usually better absorbed than guidance from the management of the facility. Collaboration with dentists and having an oral health policy are crucial for improving residents’ oral health and adherence to guidelines [31]. A study involving about 195 nursing home operators in Japan reported that nursing staff acquired dental knowledge on a case-by-case basis through interactions with residents. The findings emphasized the need for professional training to equip caregivers with the skills required to effectively manage residents’ oral health needs [32].

### 4.4. Support and Implementation of GENS-POHN

Within the GENS-POHN, personal competence is required of care givers, which is to be acquired from nursing professionals who have completed specific training for the promotion of oral health. According to the GENS-POHN, these so-called “experts for oral care” have the task of promoting knowledge transfer and correct oral care in the facility, e.g., by conducting theoretical training and practical courses. Furthermore, they are to act in an advisory capacity during visits and support the facilities in their contact with dentists [5].

The nursing staff in this study showed a positive attitude towards the GENS-POHN, considering it important, necessary, and useful. This positive attitude is in line with previous studies that have shown that nursing staff are willing to improve their knowledge and skills in oral healthcare [17,29]. Furthermore, the participants in our study expressed the hope that implementing the GENS-POHN would lead to greater safety, competence, and knowledge in their daily oral healthcare tasks. This expectation is consistent with previous studies showing that the implementation of oral healthcare guidelines or standards can improve oral health outcomes in LTCFs [5,29].

Despite their positive attitude towards the GENS-POHN, the nursing staff were neutral about how the implementation would make their work easier and save time. This result suggests that more information and support might be needed to facilitate the implementation of the GENS-POHN in LTCFs. A previous study reported that a lack of resources and support, as well as competing priorities, can hinder the implementation of oral healthcare guidelines [17]. Therefore, adequate resources and support should be provided to ensure successful implementation of the GENS-POHN.

Implementing the GENS-POHN could be a crucial step towards improving oral health outcomes in this population. Although oral healthcare needs are identified to some extent in all facilities, there are still no professional experts responsible for managing oral healthcare in these facilities, which is, however, provided for by the GENS-POHN (see above) [5].

Adequate resources for training and staff support are essential. Training programs should include both theoretical and practical elements tailored to caregivers’ varying levels of responsibility. Practical exercises supervised by experienced professionals are particularly important for nursing assistants who perform daily oral hygiene. Previous studies have demonstrated that combining theoretical and practical training can improve caregivers’ oral health knowledge and attitudes, resulting in better oral healthcare delivery [30]. Other authors also conclude that care providers should equip their staff with the necessary knowledge and skills for oral care and prioritize this area of personal care [33].

Most participants stated that they did not use oral health screening or assessment instruments in their daily oral care. This could be a disadvantage, although it is known that these assessment tools are not always reliable in terms of their validity and reliability [15]. The GENS-POHN envisages the use of a two-stage procedure, whereby a first screening is followed by a more in-depth assessment if the screening reveals oral problems. Both for screening and assessment, the GENS-POHN describes interprofessional coordinated criteria concerning oral health in nursing care.

### 4.5. Study Limitations

This study’s limitations include its small sample size and sole focus on caregivers. Future research should involve residents and their dentists, in addition to caregivers, to gain a comprehensive understanding of oral health practices in nursing homes. The long-term effectiveness of the GENS-POHN should also be evaluated.

The locations of the facilities in the study provide an interesting mix of urban and rural settings, which seems representative of Germany as a whole. Nevertheless, it should be emphasized that these are only locations in the south-western part of Germany. Other regions or other federal states with possibly different influencing factors were not included here. This could lead to a bias if the study does not take into account the full range of geographical and demographic differences in the region or country. Therefore, future studies should consider other locations with a wider regional range in order to obtain a more comprehensive perspective.

### 4.6. Need for Oral Healthcare Education and Training among Caregivers

Oral healthcare in nursing facilities could benefit from better training for non-dental healthcare professionals. Such training is crucial given the widespread poor oral health and low dental visit rates among long-term care facility residents [34,35].

At the time of admission, facilities should offer an initial dental examination as standard. This would allow for timely dental treatment and provide caregivers with essential information about new residents’ oral health.

### 4.7. Recommendations for Professional Training

Non-dental healthcare professionals could provide oral health advice, perform basic preventive measures, and refer patients to a dentist when appropriate [36].

Developing geriatric oral health education programs for non-dental healthcare professionals is recommended. Training should be provided for a range of professionals, including physicians, nurses, nursing assistants, physical therapists, occupational therapists, medical assistants, pharmacists, and dietitians [37]. This is consistent with the diverse professional backgrounds of this study’s participants, who were selected by their institutions to participate based on their day-to-day contact with oral healthcare.

Despite short-term improvements in knowledge, attitude changes have been limited; clear, evidence-based strategies are needed to ensure adherence to guidelines [38]. The introduction of oral care assistants in long-term care facilities has shown a positive impact on residents’ oral health [39]. Despite all the considerations and goodwill in this topic, it must be clear that sustainable oral care requires time and must be very individualized. The requirements of an older person versus a person who has been cognitively impaired since birth are very different. The care standard provides support for every requirement.

### 4.8. Future Directions of Research

Future research should focus on developing and implementing oral healthcare programs in nursing care facilities, with particular attention to training and the use of the GENS-POHN. Furthermore, oral hygiene education should be emphasized in nursing schools to ensure comprehensive training for nursing students. It is important to increase teacher training in this area. This is the only way to guarantee comprehensive training for nursing students, ultimately leading to better oral health outcomes for residents in nursing care facilities.

On an international level, this research could provide nurses in other countries with a means of comparing their own education and knowledge levels. Furthermore, it could serve as an impetus for the development and implementation of national nursing standards in other countries and social contexts, which should be accompanied by scientific studies during their development and implementation.

## 5. Conclusions

In conclusion, this study highlights the need for improved oral healthcare in nursing facilities and the importance of implementing the GENS-POHN. The results indicate positive attitudes towards the standard among nursing assistants but also highlight knowledge gaps and the need for further training. It is crucial to reduce these education and training deficits, and thus address oral health issues together in the practical day-to-day care setting. The goal is to achieve better oral health and thus also contribute to general health. The general well-being of the residents of care facilities would also be promoted as a result.

## Figures and Tables

**Table 1:** Socio-demographic characteristics of the study population in total and stratified by care setting (LTCF—long-term care facility) (period of training is defined as the entire period of vocational training and continuing education) (others: “not specified” (n = 2)).

		Acute Geriatric Clinic	LTCF for Young Residents	LTCF for Old Residents	Outpatient Care Facility	LTCF for People with Disabilities	Total
		n = 13	n = 28	n = 17	n = 4	n = 17	n = 79
Gender (n/%)	Male	1/7.7	6/21.4	2/11.8	1/25.0	4/23.5	14/17.7
	Female	12/92.3	22/78.6	15/88.2	3/75.0	12/70.6	64/81.0
	Not specified	0/0	0/0	0/0	0/0	1/5.9	1/1.3
Age (in years)	Mean (±SD)	42.7 ± 13.4	32.0 ± 10.8	40.1 ± 13.4	40.5 ± 7.7	39.3 ± 15.5	37.5 ± 13.1
	Median (Range)	48.5 (22–57)	29 (16–63)	44 (19–41)	37 (36–52)	32.5 (19–62)	34 (16–63)
Period of training (in years)	Mean (±SD)	3.3 ± 1.8	2.5 ± 1.5	2.1 ± 1.7	3.3 ± 0.5	2.7 ± 1.6	2.6 ± 1.6
	Median (Range)	3 (0-6)	3 (0–5.5)	3 (0–5.5)	3 (3–4)	3 (0–7)	3 (0–7)
Position in the facility (n/%)	Unit manager	0/0	0/0	0/0	2/50.0	0/0	2/2.5
	Nursing specialist	0/0	3/10.7	3/17.6	0/0	0/0	6/7.6
	Nursing assistant	6/46.2	15/53.6	7/41.2	1/25.0	10/58.8	39/49.4
	Semi-skilled labor nurse	0/0	2/7.1	3/17.6	0/0	2/11.8	7/8.9
	Trainee	2/15.4	7/25.0	3/17.6	1/25.0	4/23.5	17/21.5
	Others	5/38.4	1/3.6	1/5.9	0/0	1/5.9	8/10.1

**Table 2:** Caregivers’ knowledge of the facility’s processes, procedures, and cooperation with dentists stratified by setting and in total, and its comparison with the existing conditions stated by the facility managers (gold standard) (LTCF—long-term care facility). * Cooperation agreement does not have to be in the sense of §119b SGB V, but can also be another structured form of cooperation with a dentist.

	Acute Geriatric Clinic		LTCF for Young Residents		LTCF for Old Residents		Outpatient Care Facility		LTCF for People with Disabilities		Total
	n = 13		n = 28		n = 17		n = 4		n = 17		n = 79
	Responses of (n/%)										
	Care- Givers	Facility Manager	Care- Givers	Facility Manager	Care- Givers	Facility Manager	Care- Givers	Facility Manager	Care- Givers	Facility Manager	Care- Givers
Do you have one or more cooperation agreements * with a dentist?											
Yes	3/18.8		16/57.1	Yes	2/11.8	Yes	0/0	Yes	10/58.8		30/38.0
No	8/50.0	No	6/21.4		6/35.3		2/50.0		3/17.6		23/29.1
In preparation	0/0		1/3.6		3/17.6		1/25.0		0/0		5/6.3
I do not know	5/31.3		5/17.9		6/35.3		1/25.0		4/23.5	Do not know	21/26.6
Does your facility have a dedicated oral healthcare manager?											
Yes	5/31.3		1/3.6		0/0		0/0		3/17.6		9/11.4
No	4/25.0	No	19/67.9	No	13/76.5	No	2/50.0	No	11/64.7	No	48/60.8
I do not know	7/43.8		8/28.6		4/23.5		2/50.0		3/17.6		22/27.8
In the past two years, has your facility offered continuing education on oral health in nursing?	n=15								n=16		n=77
Yes	4/26.7		2/7.1		0/0		0/0		4/25.0		9/11.7
No	9/60.0	No	15/53.6	No	13/76.5	No	3/75.0	No	8/50.0	No	46/59.7
I do not know	2/13.3		11/39.3		4/23.5		1/25.0		4/25.0		22/28.6
When your residents/clients/patients are admitted, are they asked how they would like to receive primary medical care in the future?											
Yes	10/62.5	Yes	17/60.7	Yes	13/76.5	Yes	2/50.0	Yes	12/70.6		52/65.8
No	5/31.3		3/10.7		0/0		1/25.0		2/11.8	No	11/13.9
I do not know	1/6.3		8/28.6		4/23.5		1/25.0		3/17.6		16/20.3
When your residents/patients are admitted, are they asked how they would like to receive dental services in the future?											
Yes	1/6.3		2/7.2		2/11.8	Yes	1/25.0	Yes	5/29.4		10/12.7
No	11/68.8		13/46.4	No	10/58.8		2/50.0		4/23.5	No	40/50.6
I do not know	4/25.0	If required	14/46.4		5/29.4		1/25.0		8/47.1		29/36.7
Is a dental examination arranged upon admission of the resident/patient?											
Yes	4/25.0		5/17.9		1/5.9		0/0		6/35.3		15/19.0
No	9/56.3		14/50.0	No	10/58.8	No	3/75.0	No	5/29.4	No	41/51.9
I do not know	3/18.8	If required	9/32.1		6/35.3		1/25.0		6/35.3		23/29.1
Upon admission of your residents/patients, are nursing support needs assessed to promote oral health?											
Yes	9/56.3		11/39.3	Yes	7/41.2	Yes	1/25.0	Yes	6/35.3		32/40.5
No	3/18.8	No	5/17.9		2/11.8		0/0		1/5.9		10/12.7
Partly	4/25.0		5/17.9		3/17.6		2/50.0		7/41.2	Partly	21/26.6
I do not know	0/0		7/25.0		5/29.4		1/25.0		3/17.6		16/20.3

## Data Availability

The datasets generated and/or analyzed during the current study are not publicly available due to ethical and data safety reasons, but are available from the corresponding author upon reasonable request.

## Supplementary Materials

The following supporting information can be downloaded at: https://www.mdpi.com/article/10.3390/geriatrics9050112/s1. File S1—Detailed description of the GENS-POHN. File S2—Description of the nursing profession as defined by the current German Classification of Occupation 2010 (revised version 2020) [5]. File S3—Examined questionnaire sections in detail.

## References

[1] European Commission, Social Protection Committee (2021). 2021 Long-Term Care Report. Trends, Challenges and Opportunities in an Ageing Society.

[2] United Nations (2019). World Population Prospects 2019: Highlights.

[3] United Nations, Department of Economic and Social Affairs (2023). World Social Report 2023: Leaving No One behind in an Ageing World.

[4] Statistisches Bundesamt (destatis) Pflegestatistik (2022). Pflege Im Rahmen der Pflegeversicherung. Deutschlandergebnisse.

[5] Deutsches Netzwerk für Qualitätsentwicklung in der Pflege (DNQP) (2023). Expertenstandard zur Förderung der Mundgesundheit in der Pflege.

[6] Nitschke I., Hahnel S. (2021). Dental care for older people: Opportunities and challenges. Bundesgesundheitsblatt Gesundheitsforschung Gesundheitsschutz.

[7] Score Personal (2023). Expertenstandards in der Pflege—Eine Allgemeine Übersicht.

[8] Gati D., Vieira A.R. (2011). Elderly at Greater Risk for Root Caries: A Look at the Multifactorial Risks with Emphasis on Genetics Susceptibility. Int. J. Dent..

[9] Raphael C. (2017). Oral Health and Aging. Am. J. Public Health.

[10] Iinuma T., Arai Y., Abe Y., Takayama M., Fukumoto M., Fukui Y., Iwase T., Takebayashi T., Hirose N., Gionhaku N. (2015). Denture Wearing during Sleep Doubles the Risk of Pneumonia in the Very Elderly. J. Dent. Res..

[11] Holmstrup P., Damgaard C., Olsen I., Klinge B., Flyvbjerg A., Nielsen C.H., Hansen P.R. (2017). Comorbidity of Periodontal Disease: Two Sides of the Same Coin? An Introduction for the Clinician. J. Oral Microbiol..

[12] Hopcraft M., Tan C. (2010). Xerostomia: An Update for Clinicians: Xerostomia: An Update for Clinicians. Aust. Dent. J..

[13] Wong F.M.F., Ng Y.T.Y., Leung W.K. (2019). Oral Health and Its Associated Factors Among Older Institutionalized Residents—A Systematic Review. Int. J. Environ. Res. Public Health.

[14] Anderson R.A., Wang J., Plassman B.L., Nye K., Bunn M., Poole P.A., Drake C., Xu H., Ni Z., Wu B. (2019). Working Together to Learn New Oral Hygiene Techniques: Pilot of a Carepartner-Assisted Intervention for Persons with Cognitive Impairment. Geriatr. Nurs..

[15] Jockusch J., Hopfenmüller W., Sobotta B.A.J., Nitschke I. (2021). Interrater Reliability and Concurrent Validity of Oral/Dental Items in the Resident Assessment Instrument Minimum Data Set 2.0. Gerodontology.

[16] Zenthoefer A., Meyer-Kühling I., Hufeland A.-L., Schröder J., Cabrera T., Baumgart D., Rammelsberg P., Hassel A. (2016). Carers’ Education Improves Oral Health of Older People Suffering from Dementia &ndash; Results of an Intervention Study. CIA.

[17] Hoben M., Clarke A., Huynh K.T., Kobagi N., Kent A., Hu H., Pereira R.A.C., Xiong T., Yu K., Xiang H. (2017). Barriers and Facilitators in Providing Oral Care to Nursing Home Residents, from the Perspective of Care Aides: A Systematic Review and Meta-Analysis. Int. J. Nurs. Stud..

[18] Estes K.R., Callanan D., Rai N., Plunkett K., Brunson D., Tiwari T. (2018). Evaluation of an Interprofessional Oral Health Assessment Activity in Advanced Practice Nursing Education. J. Dent. Educ..

[19] Haber J., Hartnett E., Allen K., Hallas D., Dorsen C., Lange-Kessler J., Lloyd M., Thomas E., Wholihan D. (2015). Putting the Mouth Back in the Head: HEENT to HEENOT. Am. J. Public Health.

[20] Kassenzahnärztliche Bundesvereinigung, GKV-Spitzenverband (2019). Bericht der Kassenzahnärztlichen Bundesvereinigung und des GKV-Spitzenverbands an die Bundesregierung zur Entwicklung der Kooperativen und Koordinierten Zahnärztlichen und Pflegerischen Versorgung von Pflegebedürftigen Versicherten in stationären Pflegeeinrichtungen gem. § 119b Abs. 3 Satz 3 SGB V. https://www.gkv-spitzenverband.de/media/dokumente/krankenversicherung_1/zahnaerztliche_versorgung/zae_sonstige_vereinbarungen_1/ZA_Pflege_Bericht-bundesreg-119b-SGBV-2019-06-30-final.pdf.

[21] Sirsch E., Ludwig E., Müller K., Blumenberg P., Nitschke I., Büscher A. (2022). Förderung der Mundgesundheit in der Pflege—Ein interprofessioneller Expertenstandard. Z. Gerontol. Geriat..

[22] Deutsches Netzwerk für Qualitätsentwicklung in der Pflege (2019). Methodisches Vorgehen zur Entwicklung, Einführung und Aktualisierung von Expertenstandards in der Pflege und zur Entwicklung von Indikatoren zur Pflegequalität auf Basis von Expertenstandards. https://www.dnqp.de/fileadmin/HSOS/Homepages/DNQP/Dateien/Weitere/DNQP-Methodenpapier2019.pdf.

[23] Moers M., Schiemann M., Stehling H. (2014). Expertenstandards Implementieren—Spezifika Gelingender Einführungsprozesse. Qualitätsentwicklung in der Pflege—Konzepte, Methoden und Instrumente.

[24] Waterkotte R., Ludwig E., Nitschke I., Nitschke I., Wefers K.-P., Jockusch J. (2023). Berufsbilder in Der Pflege. Mobile Zahnmedizin. Die aufsuchende Betreuung.

[25] Bundesagentur für Arbeit (2021). Klassifikation der Berufe 2010—Überarbeitete Fassung 2020. https://statistik.arbeitsagentur.de/DE/Navigation/Grundlagen/Klassifikationen/Klassifikation-der-Berufe/KldB2010-Fassung2020/KldB2010-Fassung2020-Nav.html.

[26] Lesińska-Sawicka M. (2022). Nurses’ Attitudes towards Selected Social Groups: Cross-Sectional Survey among Polish Nurses. Healthcare.

[27] IBM (2021). SPSS Statistics for Windows.

[28] Donabedian A. (2005). Evaluating the quality of medical care. 1966. Milbank Q..

[29] Khanagar S., Kumar A., Rajanna V., Badiyani B., Jathanna V., Kini P. (2014). Oral Health Care Education and Its Effect on Caregivers′ Knowledge, Attitudes, and Practices: A Randomized Controlled Trial. J. Int. Soc. Prevent. Communit. Dent..

[30] Frenkel H., Harvey I., Needs K. (2002). Oral Health Care Education and Its Effect on Caregivers’ Knowledge and Attitudes: A Randomised Controlled Trial. Community Dent. Oral Epidemiol..

[31] Palmers E., Janssens L., Phlypo I., Vanhaecht K., De Almeida Mello J., De Visschere L., Declerck D., Duyck J. (2022). Perceptions on Oral Care Needs, Barriers, and Practices Among Managers and Staff in Long-Term Care Settings for Older People in Flanders, Belgium: A Cross-Sectional Survey. Innov. Aging.

[32] Burks C.E., Jones C.W., Braz V.A., Swor R.A., Richmond N.L., Hwang K.S., Hollowell A.G., Weaver M.A., Platts-Mills T.F. (2017). Risk Factors for Malnutrition among Older Adults in the Emergency Department: A Multicenter Study. J. Am. Nitschke Soc..

[33] Elliot V. (2017). Challenges of Improving Oral Health for Adults in Care Homes. Nurs. Older People.

[34] Sweeney M.P., Williams C., Kennedy C., Macpherson L.M.D., Turner S., Bagg J. (2007). Oral Health Care and Status of Elderly Care Home Residents in Glasgow. Community Dent. Health.

[35] Chen X., Clark J.J.J., Naorungroj S. (2013). Oral Health in Nursing Home Residents with Different Cognitive Statuses. Gerodontology.

[36] Kossioni A.E., Maggi S., Müller F., Petrovic M. (2018). Oral Health in Older People: Time for Action. Eur. Geriatr. Med..

[37] Kossioni A., McKenna G., Müller F., Schimmel M., Vanobbergen J. (2017). Higher Education in Gerodontology in European Universities. BMC Oral Health.

[38] Weening-Verbree L., Huisman-de Waal G., van Dusseldorp L., van Achterberg T., Schoonhoven L. (2013). Oral Health Care in Older People in Long Term Care Facilities: A Systematic Review of Implementation Strategies. Int. J. Nurs. Stud..

[39] Wårdh I.M., Wikström M.B. (2014). Long-term Effects of Using Oral Care Aides at a Nursing Home for Elderly Dependent Residents—A Pilot Study. Spec. Care Dent..

