# Supplementary material for: Implementation of the Expert Nursing Standard: Caregivers’ Oral Health Knowledge
Source: Geriatrics (Basel). 2024 Sep 3;9(5):112. doi: 10.3390/geriatrics9050112 (PMC11417731; doi:10.3390/geriatrics9050112)
Supplement: Supplementary file 1 [file geriatrics-09-00112-s001.zip › Supplementary File S1.pdf]

Supplementary File S1

The following presentation is a translation by the authors that provides an overview of the expert standard, which is originally only available in German. This translation was produced with the kind permission of the DNQP and is based on the document that is available in its original form at the following link: https://www.dnqp.de/fileadmin/HSOS/Homepages/DNQP/Dateien/Expertenstandards/Mundgesundheit/Mund_AV_Auszug.pdf

Title: Expert Standard for the Promotion of Oral Health in Nursing Date: August 2021

Objective: Individuals with a need for nursing support in oral care receive assistance tailored to their specific requirements and preferences in promoting oral health. The aim is to prevent diseases of the teeth and periodontium, inflammations, undesired alterations or injuries to the oral cavity and oral mucosa, as well as complications related to dental prosthetics. In cases of existing issues, efforts should be made to contribute to the improvement of oral health and the functionality of teeth and dental prosthetics, or to prevent further deterioration.

Rationale: Problems in the oral region frequently occur and can significantly impact subjective well-being and health. Through early identification of support needs in oral care, including mouth, teeth, and dental prosthetics, careful assessment of oral health and the functionality of teeth and prosthetics, as well as the planning, implementation, and evaluation of individual measures, nursing professionals contribute to enhancing oral health and well-being. This approach also acts as a preventive measure against the onset of problems.

Structure criteria	Process criteria	Result criteria
**S1a** The **caregiver** has the competence to identify the need for nursing support in oral care.<br><br>**S1b** The **facility** ensures that the necessary materials for the assessment and documentation of oral health are available. It ensures that further expertise can be called in if necessary.	**P1a** At the beginning of the care assignment, the **caregiver** carries out an initial assessment (screening) to determine whether there are any problems or risks in the oral area. The assessment is repeated at intervals to be determined for each setting and each individual.<br><br>**P1b** The **caregiver** carries out an assessment if problems are identified or expected in the oral area and calls in further expertise if necessary.	**E1** An up-to-date, systematic and target group-specific assessment of oral health is available for people who require care support in carrying out oral care or who are expected to have oral health problems.
**S2a** The **caregiver** has skills in planning and coordinating measures to promote oral health.<br><br>**S2b** The **facility** has a procedural regulation for the promotion of oral health in which the procedure, responsibilities and interfaces are specified.	**P2 The caregiver** plans measures to promote oral health together with the person with care support needs and, if applicable, their relatives and other professional groups involved in their care. Planning is based on the assessment, taking into account individual preferences, dislikes, habits and existing self-management skills.	**E2** An individual action plan is available which takes into account the current problems in the oral area, possible risks, the individual care goals and the self-management skills of the person with a need for care support when carrying out oral care.

**S3a** The **caregiver** has the competence to provide information, education and advice on the promotion of oral health.<br><br>**S3b** The **facility** provides appropriate information, training and counseling material.	**P3a** The **caregiver** informs, trains and advises the person with care support needs and, if necessary, their relatives on how to carry out oral care. In doing so, they support and promote self-management skills. The information, training and advice is provided in close coordination with the professional groups involved in the care and on the basis of the agreed objectives.<br><br>**P3b** The **caregiver** consults further expertise if there is a special need for information, training and advice.	**E3** The person with a need for nursing support in performing oral care and, if applicable, their relatives are informed, trained and advised about the importance of oral health and measures to promote it. Self-management skills for independent oral care are supported and promoted within the framework of the agreed objectives.
**S4a** The **caregiver** has the competence to implement nursing measures to promote oral health.<br><br>**S4b** The **facility** ensures that aids, materials and suitable spatial conditions are available for the provision of oral care.	**P4a** The **caregiver** carries out the care measures to promote oral health in consultation with the person with an oral care support need and, if necessary, their relatives.<br><br>**P4b** The **caregiver** coordinates the cooperation between the professional groups involved.	**E4** The measures have been coordinated with all parties involved and implemented in accordance with the action plan.
**S5** The **caregiver** has the competence to assess the achievement of individually agreed goals and the effects of nursing measures on oral health.	**P5** The **caregiver** assesses the effectiveness of nursing measures and the success of treatment on a regular and ad hoc basis using individually agreed objectives.	**E5** An evaluation of the care measures is available. The measures have had a positive effect on the oral health and self-management of people with care-related support needs when performing oral care within the framework of the agreed objectives.
