# Supplementary material for: Implementation of the Expert Nursing Standard: Caregivers’ Oral Health Knowledge
Source: Geriatrics (Basel). 2024 Sep 3;9(5):112. doi: 10.3390/geriatrics9050112 (PMC11417731; doi:10.3390/geriatrics9050112)
Supplement: Supplementary file 1 [file geriatrics-09-00112-s001.zip › Supplementary File S2.pdf]

Supplementary File S2

Description of the nursing profession as defined by the current German Classification of Occupation 2010 (revised version 2020) [4].

These professional titles are assigned to the following levels of education:

- **Level A - Head of nursing:** The person within nursing who is responsible for organizing, managing, and supervising a nursing service to ensure that quality nursing services are provided and that operational processes run smoothly. Some of them studied in various forms with diverse emphases and obtained a bachelor or master degree.
- **Level B - Nursing specialists:** Furthermore, there are graduated nurses or geriatric nurses who have completed a 3-year dual training (combination of theoretical and practical training) and are allowed to perform all nursing measures. In the present work, they will all be referred to as nursing specialists since different nursing settings will be included in the evaluation. [4] Within the group of nursing specialists the **unit manager** appears, who is responsible for a particular unit.
- **Level C - Nursing assistants:** In Germany, there are nursing assistants, who have completed a one or two-year dual training program, depending on the federal state. They are only allowed to perform a limited list of tasks in nursing. These include all activities of nursing assistants related to mobility, personal hygiene, nutrition as well as excretion, and the management of normal everyday situations. In addition, nursing assistants support the nursing specialists (see above) in treatment care. For example, nursing assistants may assist with administering medications such as eye drops, applying ointments, or putting on and taking off compression stockings. However, nursing assistants are not responsible for accompanying physicians during rounds or making further arrangements with the attending physician or dentist. [4]
- **Level D - Semi-skilled labor nurses:** Persons who have no nursing training but usually enter nursing from another profession are referred to as semi-skilled labor nurses. In both outpatient and inpatient settings, semi-skilled labor nurses support or take over the personal care of people in need of care or guide them in this task. Personal care includes washing, showering, bathing, fingernail care, hair care, and oral care. Furthermore, semi-skilled labor nurses provide support in maintaining mobility, preparing food, and assisting with the intake of the food itself. This person can also help with excretion and compensating for urinary or fecal incontinence, if necessary, as well as with coping with normal everyday situations. Due to their lack of professional qualifications, semi-skilled labor nurses are not allowed to carry out any measures ordered by a doctor, i.e., treatment nursing. [4]
- **Level E - Trainees**
