# Supplementary material for: Implementation of the Expert Nursing Standard: Caregivers’ Oral Health Knowledge
Source: Geriatrics (Basel). 2024 Sep 3;9(5):112. doi: 10.3390/geriatrics9050112 (PMC11417731; doi:10.3390/geriatrics9050112)
Supplement: Supplementary file 1 [file geriatrics-09-00112-s001.zip › Supplementary File S3.pdf]

## Supplementary File S3

Content of questionnaire sections examined:

A) Caregivers' knowledge of the facility's processes, procedures, and facility cooperation with dentists.

- Presence of cooperative agreements with dentists.
- Availability of oral health care managers.
- Availability of continuing education on oral health in nursing.
- A query of primary medical care for residents/patients in the future upon entry into the facility.
- A query of dental services for residents/patients in the future upon entry into the facility.
- Availability of dental entrance examinations.
- Assessment of nursing support needs to promote oral health at the time of admission.

B) Attitudes and hopes of nurses regarding the implementation of GENS-POHN and their assessment of the structural criteria.

- Nurses’ attitudes towards and hopes regarding the implementation of GENS-POHN.
- GENS-POHN comprises a tabular presentation in which an objective and a rationale are listed at the very top. Below these are the three columns "Structure", "Process” and "Result", in conformity with the quality model according to Avedis Donabedian. [4] No concrete action steps are offered; the implementation of one's own individual standards, which fulfill the framework conditions of GENS-POHN, is thus the task of each facility.

  "Structural quality" refers to the infrastructure as well as available professional and human resources. Specifically, in this context, prerequisites and expectations are found for the "facility" and for the "nursing professional." [6]

  In the area of "process quality", the implementation is explained. As a rule, only the "nursing specialist" is considered here. [6]

  "Outcome quality" describes an anticipated state, a goal, similar to a nursing process plan. [6]

  In the survey of caregivers evaluated here, only the structural criteria of GENS-POHN concerning caregivers (S1-S5) were queried. [4] These are:
- Assessment of oral health problems (S1).
- Planning oral health promotion activities (S2).
- Information, education, and counseling related to oral health promotion (S3).
- Implementation of nursing interventions to promote oral health (S4).
- Assessing the effectiveness of nursing interventions to promote oral health (S5).

C) Oral health and oral/denture care in practice and caregivers` awarness

- Assessment of importance of caregivers` own oral health and oral health of residents/patients.
- Assessment of importance of oral health and oral/denture care in caregiver work for the caregiver personally and within the care facility.
- Assessment of level of knowledge.
- Assessment of need for continuing education on oral health and oral/denture care for the caregiver personally and within the facility by caregivers.

D) Assessment of theoretical knowledge about oral health and oral health care

The theoretical knowledge of the caregivers is evaluated with a written multiple choice (MC) questionnaire in four categories: oral/denture care or application of care products, oral health and general diseases / diseases of the oral cavity, anatomy / physiology of oral structures, and image questions.
